# Mitigating Cold Ischemic Injury: HTK, UW and IGL-2 Solution’s Role in Enhancing Antioxidant Defence and Reducing Inflammation in Steatotic Livers

**DOI:** 10.3390/ijms25179318

**Published:** 2024-08-28

**Authors:** Raquel G. Bardallo, Gabriela Chullo, Norma Alva, Joan Rosello-Catafau, Yiliam Fundora-Suárez, Teresa Carbonell, Arnau Panisello-Rosello

**Affiliations:** 1 Department of Cell Biology, Physiology and Immunology, Faculty of Biology, Universitat de Barcelona, 08028 Barcelona, Spainnvalva@ub.edu (N.A.); tcarbonell@ub.edu (T.C.); 2 Institut d’Investigacions Biomediques August Pi i Sunyer (IDIBAPS), University of Barcelona, 08036 Barcelona, Spain; chullo@clinic.cat (G.C.); jrcbam@iibb.csic.es (J.R.-C.); fundora@clinic.cat (Y.F.-S.); 3 Experimental Pathology Department, Institut d’Investigacions Biomèdiques de Barcelona—Consejo Superior de Investigaciones Científicas (IIBB-CSIC), 08036 Barcelona, Spain

**Keywords:** steatotic liver, static cold storage, ischemic injury, preservation solutions, HTK, UW, IGL-2, oxidative stress, sterile inflammation

## Abstract

Liver transplantation remains the only definitive treatment for end-stage liver diseases. However, the increasing prevalence of fatty liver disease among potential donors exacerbates the shortage of suitable organs. This study evaluates the efficacy of the preservation solution Institut Georges Lopez-2 (IGL-2) compared to Histidine–Tryptophan–Ketoglutarate (HTK) and University of Wisconsin (UW) preservation solutions in mitigating ischemia-reperfusion injury (IRI) in steatotic livers. Using Zucker Obese rat livers, we assessed the impact of 24-h static cold storage (SCS) with each solution on transaminase release, glutathione redox balance, antioxidant enzyme activity, lipoperoxidation, and inflammation markers. IGL-2 and UW solutions demonstrated reduced transaminase and lactate levels compared to HTK, indicating better preservation of liver integrity. IGL-2 maintained a higher reduced glutathione/oxidized glutathione (GSH/GSSG) ratio, suggesting more effective management of oxidative stress. Antioxidant enzyme activities catalase, superoxide dismutase, and glutathione peroxidase (CAT, SOD, GPX) were higher in IGL-2 preserved livers, contributing to decreased oxidative damage. Lipid peroxidation markers and inflammatory markers were lower in IGL-2 than in HTK, indicating reduced oxidative stress and inflammation. Additionally, improved mitochondrial function was observed in the IGL-2 group, correlating with reduced reactive oxygen species (ROS) production and lipid peroxidation. These findings suggest that IGL-2 offers superior preservation of liver viability, reduces oxidative stress, and minimizes inflammation compared to HTK and UW solutions. By maintaining a higher ratio of reduced glutathione and antioxidant enzyme activity, IGL-2 effectively mitigates the harmful effects of ischemia-reperfusion injury. The reduced lipid peroxidation and inflammation in the IGL-2 group further underscore its potential in improving liver transplant outcomes. These results highlight the importance of optimizing preservation solutions to enhance the viability and functionality of donor organs, potentially expanding the donor pool and improving the success rates of liver transplantation. Future research should focus on refining preservation techniques and exploring additional protective agents to further improve organ preservation and transplant outcomes.

## 1. Introduction

As of today, liver transplantation remains the sole solution for end-stage liver diseases [1,2]. However, this approach is challenged by the dwindling pool of available donors, further complicated by the increasing prevalence of fatty liver disease [3], known as steatosis, among potential donors, categorizing them as marginal donors [4]. Therefore, there is a critical imperative to expand the donor pool, particularly within the cohort of marginal donors, to address this pressing medical need effectively [5].

Liver ischemia-reperfusion injury (IRI) is a complex phenomenon encountered in liver transplantation and in various medical contexts, characterized by the interruption of blood supply followed by its restoration [6] being even more harmful for steatotic livers [7]. The ischemic phase leads to a cascade of detrimental events, including ATP depletion [8], cellular oedema [9], and acidification [10,11], exacerbating tissue damage. Although the generation of ROS during reperfusion exacerbates injury, it is crucial to note that the processes driving this ROS burst and subsequent inflammation originate during ischemia [12,13]. Interestingly, current research tends to focus more on the reperfusion phase, assuming it to be the critical period for ROS-mediated damage, overlooking the insufficient understanding of the ischemic phase’s intricacies [14]. This knowledge gap underscores the importance of delving deeper into the ischemic phase to unravel its contribution to IRI comprehensively.

Under conditions of restricted blood flow, such as ischemia-reperfusion injury, cells rapidly transition to anaerobic metabolism to sustain ATP generation [15]. Anaerobic metabolism involves the production of lactic acid [16,17], which can lead to metabolic acidosis [1]. Ultimately, ATP levels decrease due to both reduced production and increased consumption, exacerbating cellular dysfunction and tissue damage during ischemia.

In the context of cellular stress and the generation of ROS, programmed cell death mechanisms are triggered, such as apoptosis or ferroptosis to counteract damage [18,19]. The execution of apoptosis is influenced by various factors, including energy and the availability of certain caspases [20,21]. When energy levels are insufficient or caspase function is compromised, the orderly progression of apoptosis can be disrupted, leading to cellular demise through necrosis instead [22,23]. Necrotic cell death results in the release of intracellular contents, including damage-associated molecular patterns (DAMPs) such as high mobility group box 1 (HMGB1) [24,25], triggering an inflammatory response [26,27,28]. This uncontrolled type of cell death results in the release of intracellular ROS by necrotic cells, perpetuating cell death and tissue damage.

Once the inflammatory cascade is initiated, ROS can interact with various cellular components [29], particularly lipids, leading to the formation of lipoperoxides [30] and triggering ferroptosis [2]. In this context, the mitochondrial status not only influences the quantity of ROS generated but also impacts the functionality of mitochondrial enzymes such as aldehyde dehydrogenase 2 (ALDH2) [31]. ALDH2 plays a crucial role in inhibiting the accumulation of lipid peroxidation products like 4-hydroxynonenal (4-HNE) or malondialdehyde (MDA), thereby modulating the extent of oxidative damage within the cell [32]. Understanding the interplay between ROS, lipid peroxidation, and mitochondrial enzyme activity sheds light on the mechanisms underlying oxidative stress-induced cellular injury [33].

In the context of ischemia and oxidative stress, antioxidants play a pivotal role in cellular antioxidant defence mechanisms [34]. Antioxidants, such as glutathione, scavenge ROS [35], protecting cellular components from oxidative damage [36]. During periods of ischemia, the balance between the antioxidant GSH and oxidized GSSG forms of glutathione becomes disrupted, leading to a decrease in the GSH/GSSG ratio [37]. This shift toward a more oxidized state compromises the cell’s ability to combat oxidative stress effectively [38] where NADPH is needed to recover GSH from GSSG [3].

Additionally, glutathione S-transferases (GSTs), a family of enzymes that catalyse the conjugation of GSH to various electrophilic compounds, contribute to the detoxification of reactive intermediates generated during ischemia-reperfusion injury [39]. Understanding the dynamic interplay between glutathione redox balance and GST activity provides valuable insights into the mechanisms underlying cellular responses to oxidative stress during ischemic conditions.

In the field of organ preservation, despite the availability of advanced perfusion machine strategies, such as hypothermic oxygenated perfusion (HOPE) and normothermic machine perfusion (NMP), the static cold storage SCS method remains the most widely utilized due to its cost-effectiveness and logistical simplicity [40,41,42]. However, this reliance on SCS has led to a stagnation in the innovation of organ preservation solutions in recent years. Notably, one of the largest liver studies in Europe concluded that the most commonly used solutions were UW, Celsior, HTK, and IGL [43]. Consequently, there is a pressing need for a comprehensive review of the latest preservation solutions from a fresh perspective. By critically evaluating newly emerged preservation solutions, we can potentially uncover novel strategies to enhance organ viability, extend preservation times, and improve transplant outcomes. This renewed scrutiny aims to invigorate the field of organ preservation and pave the way for advancements in organ transplantation practices [44].

Effective preservation solutions play a pivotal role in maintaining the viability and functionality of harvested organs during transplantation procedures (Table 1). These solutions are meticulously formulated to sustain cellular integrity and metabolic activity, thus mitigating ischemic injury and enhancing graft survival [45,46,47]. Understanding the properties of preservation solutions is crucial for optimizing their efficacy in clinical settings, since the different components will interact and regulate with different pathways, even though the final result might be similar [4]. In this regard, physiochemical key attributes such as pH, viscosity, osmolarity, and the influence of oncotic agents are of paramount importance [46].

In this study, we provide a comparative appraisal of commonly used liver preservation solutions in liver transplantation settings, offering a fresh perspective by critically evaluating emerging solutions such as IGL-2 against established ones like HTK and UW. We aim to explore new insights by examining the direct integration of static hypothermic preservation strategies with innovative dynamic perfusion strategies such as HOPE to enhance liver transplantation outcomes. This renewed scrutiny seeks to invigorate the field of organ preservation and pave the way for advancements in transplantation practices [44].

## 2. Results

**Figure 1:**
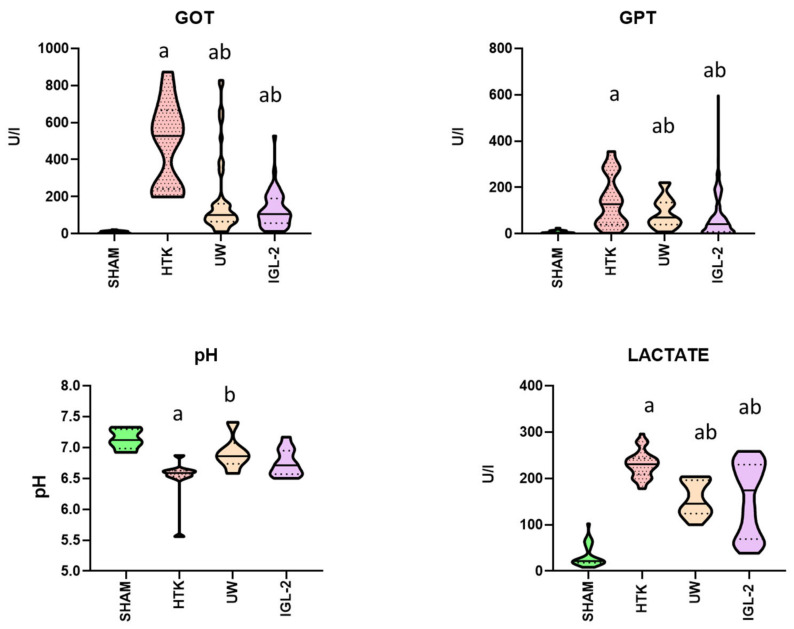
Relationships among transaminase activity (GOT, GPT), pH levels, and lactate concentration. This figure shows the release of transaminases (GOT and GPT), lactate levels, and pH in perfusates from fatty livers subjected to 24-h cold storage in IGL-2, HTK, and UW solutions. Control group without ischemia (SHAM) included. Results expressed as mean ± SEM. a *p* < 0.5 vs. SHAM, b *p* < 0.5 vs. HTK.

A significant increase in transaminases and lactate levels was observed in the perfusate from livers preserved with the HTK preservation solution, distinguishing this group statistically from livers preserved with preservation solutions containing an oncotic agent (UW and IGL-2). Compared to the SHAM control group, all groups exhibited elevated transaminases, reflecting ischemic damage. Regarding pH, a more pronounced acidification was observed in the HTK group, which could be related to the increase in lactate (Figure 1).

It is well documented that ischemia involves a decrease in ATP levels [8], which results in necrosis and the release of intracellular components that are recognized as DAMPs, subsequently impacting sterile inflammation. We analysed DAMPs (uric acid, HMGB1) [48], inflammasome components absent in melanoma-2 and nucleotide-binding oligomerization domain (nod)-like receptor protein-3 (AIM2 and NLRP3), as well as the anti-inflammatory cytokine interleukin-6 (IL-6) [49,50].

**Figure 2:**
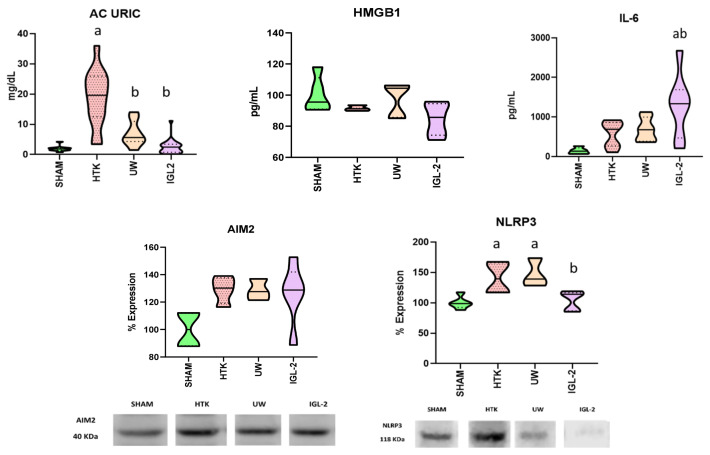
Association of uric acid, HMGB1, IL-6, AIM2, and NLRP3 levels in inflammatory responses. DAMPs measured as HMGB1 and acid uric, inflammasome, measured as AIM2 and NLRP3 and inflammatory related cytokines measured as IL-6 levels were assessed. Results expressed as mean ± SEM. a *p* < 0.5 vs. SHAM, b *p* < 0.5 vs. HTK.

Figure 2 shows a significant increase in uric acid levels in the HTK group, distinguishing it from the groups with oncotic support (UW and IGL-2), following the same pattern observed with transaminases and lactate. Regarding HMGB1, although no significant differences were observed between the groups, there was a downward trend in the IGL-2 group. For inflammasomes, no differences were seen between groups in AIM2; however, a lower concentration of NLRP3 was observed in the IGL-2 group compared to the other ischemic groups. Regarding the anti-inflammatory cytokine IL-6, we observed an increased concentration in the perfusate of livers preserved with IGL-2 compared to HTK and UW.

It is well-documented that an increase in DAMPs initiates a sterile inflammatory cascade, leading to an elevation in ROS, which in turn exacerbates inflammation [51]. In this context, we analysed GRP78 as a marker for endoplasmic reticulum stress, as well as oxidation-derived products. Additionally, we examined the mitochondrial enzyme ALDH2, responsible for detoxifying certain lipid peroxides such as 4-HNE.

**Figure 3:**
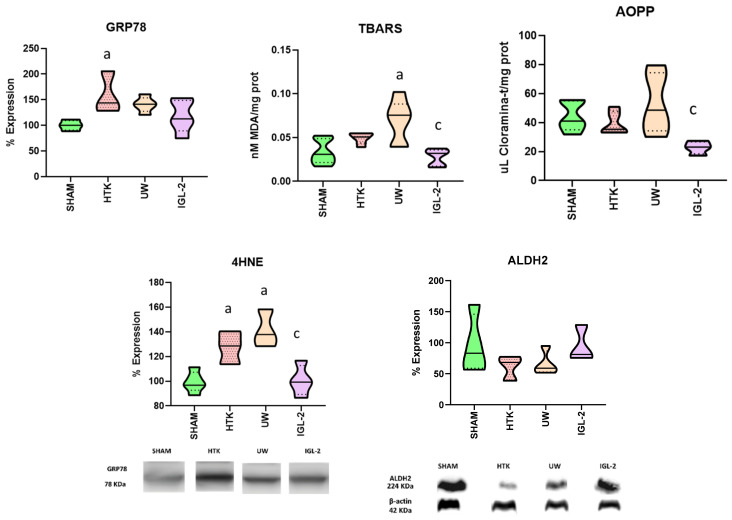
Correlation between GRP78, oxidative stress markers (TBARS, AOPP, 4HNE), and ALDH2 activity. Assessment of endoplasmic reticulum stress (GRP78), lipoperoxidation (TBARS, AOPP, 4HNE) and lipoperoxidation sub-products antagonists (ALDH2) were analysed. Results expressed as mean ± SEM. a *p* < 0.5 vs. SHAM, c *p* < 0.5 vs. UW.

We observed an increase in ERS in the HTK group compared to UW and IGL-2. Despite this, we observed higher lipoperoxidation in the UW group compared to the others, with the IGL-2 group showing the least lipoperoxidation among the ischemic groups for the markers TBARS, AOPP, and 4-HNE. Regarding the latter marker, we noted that its antagonist, the mitochondrial enzyme ALDH2, although not significantly different from the other groups, showed an upward trend in the IGL-2 group (Figure 3).

Given that labile glutathione is present as an antioxidant additive in all solutions except HTK, we evaluated the disruption in the balance between reduced glutathione (GSH) and oxidized glutathione (GSSG) and glutathione reductase (GR) after this 24-h cold ischemic period.

**Figure 4:**
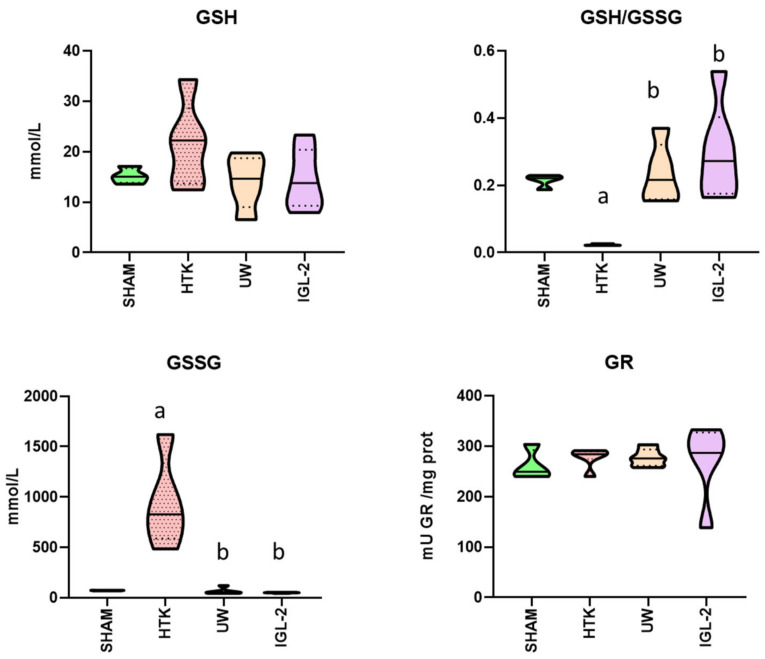
Interaction between glutathione levels (GSH, GSSG), glutathione reductase (GR) activity, and the GSH/GSSG ratio. Reduced glutathione (GSH), oxidized glutathione (GSSH), it’s ratio and glutathione reductase (GR) levels were assessed. Results expressed as mean ± SEM. a *p* < 0.5 vs. SHAM, b *p* < 0.5 vs. HTK.

Figure 4 illustrates higher levels of both GSH and GSSG in HTK compared to the other groups, consistent with lower GSH/GSSG ratio values in HTK relative to the other solutions. No alterations in glutathione reductase (GR) activity were observed.

Given that we analysed glutathione, we also examined the activities of superoxide dismutase (SOD), catalase (CAT), glutathione S-transferase (GST), and glutathione peroxidase (GPx) since these enzymes collectively play crucial roles in the cellular defence against oxidative stress and the maintenance of cellular redox.

**Figure 5:**
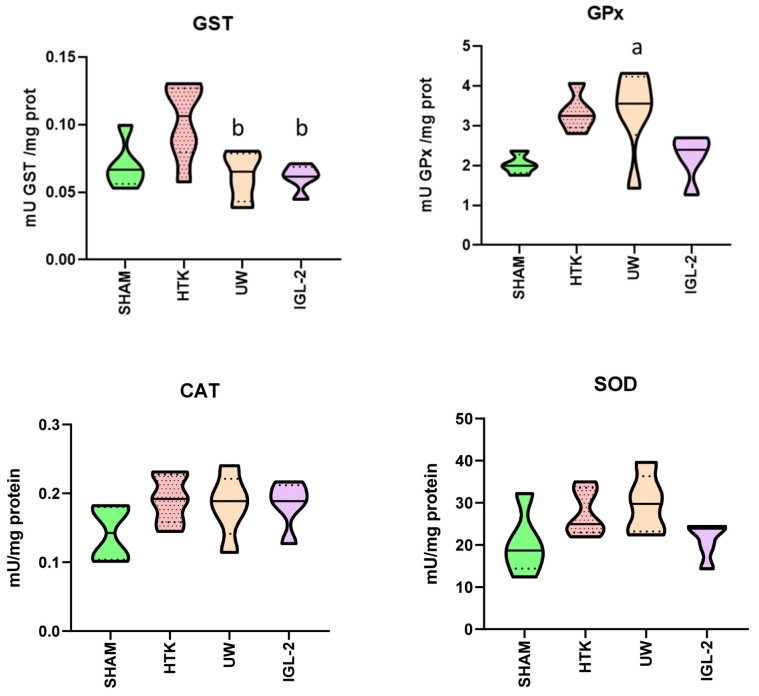
Comparison of enzymatic antioxidant activity: GST, GPx, CAT, and SOD levels. Evaluation of alterations in catalase (CAT), superoxide dismutase (SOD), glutathione S-transferase (GST), and glutathione peroxidase (GPX) enzyme profiles involved in other antioxidant mechanisms. Results expressed as mean ± SEM. a *p* < 0.5 vs. SHAM, b *p* < 0.5 vs. HTK.

We observed lower levels of GST in the UW and IGL-2 groups compared to HTK. These lower levels were maintained only in the IGL-2 group for GPx. Although no significant differences were observed between the groups for CAT and SOD, there was a downward trend in the IGL-2 group for SOD (Figure 5).

## 3. Discussion

One of the initial observations is the evident reflection of ischemic damage across all groups (with the SHAM group serving as the non-ischemic control), manifested by an increase in transaminases. Nevertheless, within the ischemia-exposed liver groups, it is noted that this elevation in transaminases (indicative of injury) is less pronounced in those preserved with solutions containing an oncotic agent in their composition, such as UW and IGL-2 solutions. This pattern similarly holds true regarding lactate presence in the perfusate. Specifically, UW and IGL-2 solutions appear to confer greater protection compared to HTK solution based on transaminase parameters and lactate.

It is widely reported that under conditions of cellular stress, cells activate cytoprotective mechanisms [52,53]. If these mechanisms fail to counteract damage, programmed cell death or apoptosis is initiated [18]. Apoptosis is regulated by accessibility to caspases and ATP [20,21]. In the absence of either component, programmed cell death cannot occur, leading instead to necrosis or ferroptosis, forms of non-programmed cell death characterized by cellular membrane destabilization, rupture, and release of intracellular contents [22,23], recognized as Damage Associated Molecular Patterns (DAMPs), thereby initiating an inflammatory cascade [24].

In the results, a decrease in DAMP levels is observed in the IGL-2 group, including HMGB1 and cytosolic uric acid, which are notably elevated in the HTK group. This reduction observed in the IGL-2 group could be partly attributed to higher ATP levels, which favour more controlled cell death processes. Regarding uric acid levels in both UW and IGL-2 groups, this could be linked to improved osmoregulation due to oncotic pressure, preventing cellular swelling and subsequent rupture, thereby mitigating inflammatory contributions.

This reduced inflammation is reflected by a decrease in the NLRP3 inflammasome in the IGL-2 group, whereas AIM2 levels remain unchanged across all groups except for the non-ischemic control group (SHAM). This finding corroborates previous studies indicating that the NLRP3 inflammasome predominates during the ischemic phase [48]. Therefore, NLRP3 serves as a potential target for intervention and a candidate for evaluating liver status as a parameter derived from primary damage, aiding in accurate liver assessment, especially in temporal studies such as dynamic perfusion.

Regarding IL-6, despite its classification in much literature as a pro-inflammatory cytokine, its role is multifaceted. Under ischemic conditions, IL-6 acts in an anti-inflammatory manner by inhibiting TNF-alpha [49] and promoting HIF expression [50]. In this context, IL-6 results align with those of NLRP3, depicting a less inflammatory profile in livers preserved with IGL-2 compared to other groups. This inflammatory response will impact tissue damage and will be pivotal during reperfusion, thus necessitating careful consideration.

A sustained inflammatory response can perpetuate continuous oxidative stress, creating a vicious cycle where ROS and inflammation mutually exacerbate each other [51]. For instance, oxidative damage can activate immune cells that, in turn, generate more ROS. Thus, one approach to mitigate inflammation is by reducing ROS levels, which interact with a wide array of cellular components. In this context, we observe that in the preservation solution, all glutathione in the HTK group exists in its oxidized form and has not been regenerated. The regeneration of reduced glutathione (GSH) from oxidized glutathione disulfide (GSSG) via glutathione reductase (GR) requires NADPH. While NADPH regeneration is not ATP-dependent, it relies on glucose-6-phosphate, a substrate also utilized to ultimately perform lactic fermentation [54]. Therefore, in situations where mitochondria are dysfunctional and there is a competition for the same substrate [54], cells can prioritize lactic fermentation for energy production and NADPH regeneration becomes compromised, consequently affecting GSH regeneration from GSSG [54], as observed in the HTK group.

The GSH/GSSG ratio is higher in groups preserved with oncotic support solutions (UW and IGL-2); however, there is no corresponding increase in glutathione reductase (GR) activity among the different groups. This suggests that reduced glutathione levels (GSH) may already be sufficient for cellular ROS levels in groups where the GSH/GSSG ratio is elevated, or that other cellular pathways are prioritized, possibly diverting the pentose phosphate pathway towards alternative functions. The former hypothesis is supported by the levels of GPx observed in the IGL-2 solution (catalysing the reaction of GSH with oxygen radicals), and further corroborated by the decreased activity of other enzymes involved in oxygen radical detoxification in the same group (CAT, SOD, GST).

One of the primary sources of ROS is mitochondrial dysfunction, resulting in upregulation of GRP78, a component of the endoplasmic reticulum stress (ERS) pathway closely associated with ROS production. These ROS contribute to an inflammatory cascade, generating additional ROS from Kupffer cells [48], perpetuating tissue damage. ROS are highly damaging and can interact with various cellular components, including lipids, leading to lipid peroxidation [30]. This is evidenced by elevated levels of MDA, AOPP, and 4HNE in the HTK and UW groups compared to IGL-2. Increased lipid peroxidation levels contribute to tissue damage and serve as markers of ROS activity. However, the lower levels observed in the IGL-2 group may not only be due to reduced ROS production but also to enhanced activity of hepatic mitochondrial detoxifying enzymes such as ALDH2. ALDH2 plays a crucial role in various liver detoxification pathways, including the detoxification of 4HNE [33]. In the IGL-2 group, higher ALDH2 activity suggests improved mitochondrial function, which may contribute to these observed differences.

Osmolarity regulates the movement of water and solutes across cell membranes, thereby influencing cellular volume and osmotic equilibrium [55]. Preservation solutions are isotonic to prevent osmotic imbalances that could cause cellular swelling or shrinkage, both of which are detrimental to cellular viability [46]. Colloid osmotic pressure, also known as oncotic pressure, exerted by oncotic agents such as hydroxyethyl starch (HES) (present in UW solution) or polyethylene glycol 35 (PEG35) (present in IGL-2 solution), is a key factor that influences the movement of fluids across capillary walls, preventing oedema [56]. It is primarily determined by the concentration of macromolecules, such as plasma proteins, within the vascular system [57]. Notably, the composition and concentration of oncotic agents can impact solution viscosity and osmolarity, necessitating careful consideration during formulation [58], also including the presence of antioxidants and other additives (Table 1).

An oncotic agent plays a crucial role in regulating oncotic pressure within blood vessels. Oncotic agents increase oncotic pressure, which helps to maintain fluid within capillary vessels and prevents it from leaking into surrounding tissues [56]. This prevents oedema, facilitates the reabsorption of fluid from tissues and extravascular spaces back into blood vessels, and aids in perfusion. Oncotic agents are fundamental for regulating vascular hydrodynamics [59].

**Scheme 1:**
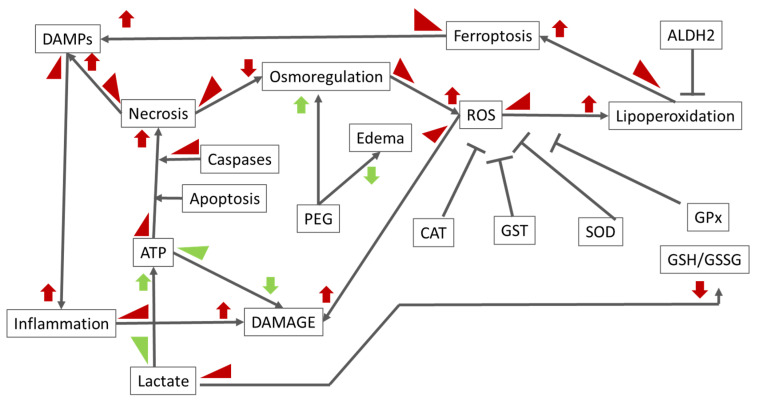
The scheme illustrates the interconnections among various biochemical pathways. Increases and decreases in compound levels are depicted at the respective points of their occurrence (beginning of grey arrows) with increment shape, while thick arrows indicate the influence (pointing upwards, increasing, pointing downwards, decreasing) on subsequent pathways at the end of the grey arrows. Negative impacts on overall cellular damage are highlighted in red, whereas beneficial cytoprotective effects are shown in green. In the scheme we observe that an increase in ROS is associated with greater damage, while an increase in ATP is associated with its reduction.

Oncotic pressure is closely related to cellular death mechanisms, including apoptosis and necrosis (Scheme 1) [46]. Modulation of oncotic pressure gradients within tissues can influence the prevalence of necrotic cell death under various pathological conditions. Increased oncotic pressure associated with extravasation of hyperosmolar solutions can lead to tissue necrosis through cellular dehydration, whereas decreased oncotic pressure due to hypoosmolar solutions may result in necrosis due to cellular oedema [60]. In this context, the findings regarding elevated DAMPs levels in the HTK perfusate group compared to UW and IGL-2 groups can be understood, since low energetic levels compromise the execution of a programmed and silent death such as apoptosis, which in turn triggers necrosis. Furthermore, low oncotic pressure in HTK enhances membrane destabilization, releasing more DAMPs. The interconnections between those pathways are shown in Scheme 1.

## 4. Material and Methods

### 4.1. Animals

Homozygous (Obese, Ob) male Zucker rats aged 11 weeks were purchased from Charles River (Charles River, Lyon, France). Zucker rats constitute a well-characterized model of nutritionally induced obesity. Steatosis in Zucker rats is not associated with inflammation, as in other models of steatosis using ethanol ingestion or a choline-deficient diet. Homozygous Zucker rats (Obese, Ob) lack the cerebral leptin receptor and develop obesity at the age of 8 weeks because of markedly increased food intake and decreased energy expenditure. They were housed in a temperature-controlled environment (25 °C) with a 12 h light/dark cycle and provided with water and standard chow ad libitum. The rats were considered ready for the experimental procedure when they reached a weight of 400 ± 20 g. The experimental procedures were conducted in accordance with protocols approved on 14 July 2016 (approval number 483116). The study adhered to the European Union Directive (EU guideline 86/609/EEC) for animal experimentation and received approval from the Ethics Committees for Animal Experimentation of the University of Barcelona (Directive 483/16). The animals were randomly assigned to the experimental groups as outlined below.

### 4.2. Experimental Groups

Group 1 (SHAM; *n* = 6): Animals underwent a transverse laparotomy, during which silk ligatures were placed in the right suprarenal vein, diaphragmatic vein, and hepatic artery. Livers were washed with 20 mL of Ringer lactate solution, and samples were collected from the flush. Tissue liver samples were collected immediately after the perfusate was collected and stored at −80 °C for further biochemical analyses, minimizing warm ischemic time to a negligible level.

Group 2 (HTK; *n* = 6): After organ recovery, the livers were flushed with 50 mL of HTK solution and stored in HTK preservation solution at 4 °C for 24 h.

Group 3 (UW; *n* = 6): After organ recovery, the livers were flushed with 50 mL of UW solution and stored in UW preservation solution at 4 °C for 24 h.

Group 4. (IGL-2; *n* = 6): After organ recovery, the livers were flushed with 50 mL of IGL-2 solution and stored in IGL-2 preservation solution at 4 °C for 24 h.

Following cold storage (4 °C), liver specimens preserved in IGL-2, UW, and HTK solutions were washed with 20 mL of Ringer lactate solution, and samples were collected from the flush. To minimize warm ischemic time, liver samples were collected immediately and stored at −80 °C for further biochemical analyses.

### 4.3. Preservation Solutions

The composition of preservation solutions is detailed in Table 1 according to Pedro-Ramos et al.’s studies [61]. The manuscript references the use of the following preservation solutions:

HTK Solution (Custodiol^®^): This solution was purchased as a ready product from Hospital Clínic de Barcelona, located at Carrer de Villarroel, 170, Barcelona, Spain.

UW Solution (Bel-Gen^®^): This solution was obtained from Institut Georges Lopez (IGL), SAS, located at Parc Tertiaire du Bois Dieu, RN6—1 allée des Chevreuils, Lissieu, France.

IGL-2 Solution^®^: This solution was also acquired from Institut Georges Lopez (IGL), SAS, located at Parc Tertiaire du Bois Dieu, RN6—1 allée des Chevreuils, Lissieu, France.

### 4.4. Biochemical Assays

GPT, GOT, Uric Acid, and Lactate: Hepatic injuries were assessed in the perfusate by quantifying levels of alanine aminotransferase (GPT) (Ref. GN40125), aspartate aminotransferase (GOT) (Ref. GN41125), uric acid (Ref. GNV10125), and lactate (Ref. GN55125) using commercial kits from RAL (Avinguda de la Mare de Déu de Montserrat, 51, Sant Joan Despí, Barcelona, Spain), following the manufacturer’s instructions.

pH Measurement: The pH of the perfusate was determined using a Crison Basic 20+ pH meter.

IL-6 and HMGB1: IL-6 and HMGB1 levels in the perfusate were analysed using Labclinics (C. de la Indústria, 54, Gràcia, Barcelona, Spain) assay kits, with references ER0042 and ER0291, respectively.

Lipid and Protein Oxidation: Lipid peroxidation was evaluated in liver tissue using the Thiobarbituric Acid Reactive Substances (TBARS) assay, detecting malondialdehyde (MDA) as a product. Liver tissues were homogenized (10% *w*/*v*) in RIPA buffer (Tris-HCl 50 mM, NaCl 150 mM, NaF 5 mM, SDS 0.1%, Triton X-100 1%, DOC 1%, pH 7.4). MDA-TBA adducts were fluorometrically measured (excitation 515 nm, emission 550 nm) with tetraethoxypropane as the standard. Results are expressed as nmol MDA-TBA adducts per mg protein. Protein oxidation was assessed by advanced oxidative protein products (AOPP) assay based on the Witko-Sarsat method. The AOPP content in liver homogenates was spectrophotometrically quantified at 340 nm, expressed as μmol Chloramine-T equivalents per mg protein. Carbonyl groups in proteins, indicative of oxidative modification, were derivatized to 2,4-dinitrophenylhydrazone (DNP hydrazone) using 2,4-dinitrophenylhydrazine (DNPH).

Hydroxynonenal Protein Adducts Assay: 4-Hydroxynonenal (4-HNE) protein adducts were measured in liver homogenate using the OxiSelect™ HNE Adduct Competitive ELISA Kit (7758 Arjons Dr, San Diego, CA, USA). Liver was homogenized in 10% (*w*/*v*) with a Teflon bar in a RIPA solution, (Tris 50 M pH 7.4, 1% Triton 100⇥, NaCl 150 mM, NaF 5 M, 0.1% sodium dodecyl sulphate, and 1% sodium deoxycholate) with antiprotease solution (aprotinin at 1.7 mg/mL, 2 μg/mL pepstatin, 2 μg/mL leupeptin and 1 mM phenylmethylsulfonyl fluoride, and sodium orthovanadate at 1 mM). The suspension was centrifuged at 2000× *g* for 5 min and the pellet discarded. Liver homogenates were added to an HNE conjugate preabsorbed ELISA plate. After 1 h incubation, an anti-HNE polyclonal antibody was added, followed by an HRP conjugated secondary antibody. The quantity of HNE adduct in protein samples was determined by comparing its absorbance with that of a known HNE-BSA standard curve.

Antioxidant Enzyme Activity: Antioxidant enzymes—superoxide dismutase (SOD, EC 1.15.1.1), glutathione S-transferase (GSH S-T, EC 2.5.1.18), glutathione peroxidase (GSH-Px, EC1.11.1), glutathione reductase (GSH-R, EC 1.6.4.2), and catalase—were measured in liver tissue with the Sigma-Aldrich Determination Kit (SOD, Cat. 19160, Sigma, St. Louis, MO, USA) and CaymanKits (GSH S-T, 703302; GSH-Px, 703102; GSH-R, 703202, Cayman Chemical, Ann Arbor, MI, USA), following the manufacturer’s instructions. Catalase activity was determined spectrometrically at 240 nm following the Aebi method (1984). Enzyme activities are reported as units (U) per mg total protein.

Glutathione (GSH) Analysis: Reduced (GSH) and oxidized (GSSG) glutathione, along with the redox ratio (GSH/GSSG), were quantified following a modified protocol of Hissin and Hilf [62]. GSH was measured fluorometrically at 350 nm (excitation) and 420 nm (emission) using ophthaldehyde (OPA) reagent. GSSG was determined after prior incubation with N-ethylmaleimide to prevent interference. Results are expressed as nmol GSH per g fresh of liver tissue or as the GSH/GSSG ratio.

Western Blotting and Quantification: Western blot analysis was performed to detect NLRP3 (Santa Cruz Biotechnology, Bergheimer Str. 89-2, Heidelberg, Germany) ALDH2 (Abcam, Biomedical Campus, Discovery Dr, Trumpington, Cambridge CB2 0AX UK. ref: ab133306), GRP78 (GRP78, Abcam ab21685) and AIM2 (Santa Cruz Biotechnology sc-293174). Liver samples were homogenized in RIPA buffer and the total protein content in liver samples (μg/mL) was determined using the Bio-Rad protein assay based on the Bradford dye-binding method. Samples were separated by SDS-PAGE, and transferred to PVDF membranes. Detection employed chemiluminescence or fluorescence, with quantification performed using the LI-COR Odyssey system and Image Studio software version 6.0. Either REVERT-TM total protein stain or β-actin were used as a loading control.

### 4.5. Statistical Analysis

All statistical analyses were performed using GraphPad Prism software version 8.0.2 (GraphPad Software, 225 Franklin Street, Fl. 26, Boston, MA, USA). Data were expressed as mean ± standard error of the mean (SEM). A one-way analysis of variance (ANOVA) was conducted to assess the statistical significance of differences among groups. Following a significant ANOVA result, post-hoc comparisons were carried out using Tukey’s test for multiple comparisons. Statistical significance was denoted as follows: ‘a’ indicates a significant difference compared to the SHAM group, ‘b’ indicates a significant difference compared to the HTK group, and ‘c’ indicates a significant difference compared to the UW group. A *p*-value of less than 0.05 was considered statistically significant. Box and violin plots were used to illustrate data distribution. The violin plot’s shape indicates the data’s density and distribution, with wider sections representing higher frequencies of data points.

## 5. Conclusions

Oncotic factor solutions yield better results in preserving the complex pathways responsible for ischemic damage in steatotic livers (See Scheme 1). However, solutions with different oncotic agents, while showing similar outcomes concerning general and traditional damage markers such as transaminases and lactate, appear to act through distinct molecular pathways, as suggested by decreased lipid peroxidation and anti-inflammatory results in IGL-2 compared to UW. It is likely that we are approaching the limit of antioxidants applicable during the ischemic phase. The interaction of these different pathways and their influence on the solutions will determine the outcome of the graft when substrates compete. In any case, IGL-2 preservation solution demonstrates superior anti-inflammatory and antioxidant properties.

## 6. Future Perspectives

As an endpoint study, this research has limitations in analysing markers over time and their metabolism, or an increase/decrease in relation to viability parameters. Therefore, given the promising results from new preservation solutions, their study should be extended to dynamic systems such as HOPE, using the same solution. Future research should investigate both systems concurrently and over time, building on the knowledge gained from static preservation studies to gain a deeper understanding of ischemic damage and solutions, ultimately providing a more optimal answer to IRI.

## Figures and Tables

**Table 1:** Composition of the preservation solutions HTK, UW, and IGL-2.

	HTK	UW	IGL-2
pH	7.02–7.2	7.4	7.4
Osmolarity, mOsm/L	310	320	360
Viscosity, cP	1.8	5.70	1.7
Colloids	–	50 g/L HES	5 g/L PEG
Impermeants			
Lactobionate mmol/L:	–	100	100
Mannitol mmol/L:	30	–	60
Raffinose mmol/L:		30	–
Buffers	Histidine	Phosphate, Sulfate	HEPES, Histidine, Phosphate, Sulfate
Electrolytes			
Calcium mmol/L:	0.015	–	–
Chloride mmol/L:	50	–	–
Magnesium mmol/L:	4	5	5
Potassium mmol/L:	10	120	25
Sodium mmol/L:	15	25	125
Zinc mmol/L:	–	–	0.091
Metabolic Precursors	α-ketoglutarate	Adenosine	Adenosine, sodium nitrite
Antioxidants and Free-Radical Scavengers	Tryptophan	Allopurinol, glutathione	Glutathione

## Data Availability

Data is contained within the article.
